# The effect of training intensity on implicit learning rates in schizophrenia

**DOI:** 10.1038/s41598-021-85686-5

**Published:** 2021-03-22

**Authors:** Natasza D. Orlov, Jessica Sanderson, Syed Ali Muqtadir, Anastasia K. Kalpakidou, Panayiota G. Michalopoulou, Jie Lu, Sukhi S. Shergill

**Affiliations:** 1 Cognition Imaging Schizophrenia Lab, Department of Psychosis Studies, Institute of Psychiatry Psychology and Neuroscience, King’s College London, London, UK; 2 Harvard Medical School, Athinoula Martinos Center for Biomedical Imaging, Massachusetts General Hospital, Boston, USA; 3 Department of Radiology, Xuanwu Hospital, Capital Medical University, Beijing, China; 4 Precision Brain Imaging Lab, Department of Neuroscience, Medical University of South Carolina, Charleston, USA; 5 Lahore University of Management Sciences, Lahore, Pakistan; 6 Marie Curie Palliative Care Research Department, University College London, London, UK

**Keywords:** Learning and memory, Schizophrenia, Cognitive neuroscience

## Abstract

Cognitive impairments in learning and memory are core symptoms of schizophrenia, associated with reduced self-reported quality of life. The most effective treatment of cognitive impairments is drill and practice cognitive training. Still, to date no study has investigated the effect of varying the frequency of training on cognitive outcomes. Here we utilized a verbal memory based language learning task, tapping into implicit cognitive processes, to investigate the role of training intensity on learning rates in individuals with schizophrenia. Data from 47 participants across two studies was utilized, one with a daily training regimen over 5 days and the other with a more intensive schedule of 5 sessions delivered over 2 days. The primary outcome measure was the change in implicit learning performance across five sessions, quantified with the Matthews Correlation Coefficient (MCC). Participants in the daily training group showed improved performance compared to the intensive group only at session 4. This is the first study to show that implicit learning rates are influenced by training intensity, with daily sessions outperforming a more intensive regimen; a period of consolidation overnight may be necessary to optimize cognitive training for individuals with schizophrenia.

## Introduction

Schizophrenia is a complex disorder, associated with positive (hallucinations and delusions), negative (blunted affect and emotional withdrawal) and cognitive symptoms (deficits in learning and memory)^1^. Of these, cognitive symptoms, or deficits, are the most debilitating in terms of clinical and functional outcomes such as medication adherence, self-reported quality of life, employment, social functioning and ability to live independently^2–5^. Cognitive deficits (CD), present in more than 70% of individuals with schizophrenia, include impairments in learning, memory, executive functions, and are largely refractive to current pharmacological treatments^6–9^. To date, the most effective treatments involve practice and drill exercises, including computerized training, with the goal of improving functioning. However, even such training only shows small to medium effect sizes in improving CD^10,11^.

Meta-analytic work suggests that the cognitive training response is associated with characteristics that can be broadly grouped into cognitive, psychological and biological factors, and may include among others, cognitive factors such as baseline cognitive performance^12^, psychological factors including motivation^13^, biological factors such as age^14^ and genetic and familial predisposition^15^.

Since many of these characteristics, such as age or genetic predisposition, are non-amenable, there is an urgent need to investigate factors that can be therapeutically impacted such as treatment regimen characteristics that demonstrate highest learning and retention rate as well as acceptability and convenience in terms of patient adherence and service costs. Most current work in cognitive remediation is to identify better strategies of treatment implementation, often in conjunction with additional therapies, such as brain stimulation or cognitive enhancing medication^8,16,17^. However, research on the effect of training intensity on learning rates and retention in schizophrenia is still lacking.

Among learning, implicit processing is a fundamentally important process in everyday life, as it involves acquiring information from the environment unconsciously^18^. It can encompass a range of motor, cognitive and social skills, including complex motor sequences, decision making in social context, ability to communicate emotions, predicting behavior and enabling individuals to adjust behavior based upon cues^18^. The number of studies examining implicit learning and memory, a core neuropsychological deficit in schizophrenia is limited. These studies conclude with variable results; some describe significant impairments in implicit processing, as in implicit associative learning, while others suggest that these processes remain intact but require greater repetition to match the performance of healthy controls, especially motor and rule judgment implicit learning^19–24^.

Learning based plastic changes occur only when a cognitive training task is practiced at a sufficient level of difficulty for the participant, and performed for a sufficient duration of time^25^. This may be of increased importance when training an impaired brain^26^. Typically, the learning is initially rapid and then slows to moderate gains in performance each session^11,26,27^.

Implicit learning and memory assessment usually involve repetition and priming, which includes identification, generation or verification of learnt stimuli relative to newly presented stimuli^28–30^. This is done without explicit instructions, as this has been found to be counterproductive^31^. From a methodological perspective the lack of instructions and explicit hypothesis testing is advantageous, as such learning is less affected by learning strategies^32^.

These characteristics make implicit learning and memory particularly suitable to investigate aspects of cognitive training regimen in individuals with schizophrenia, specifically delivery rates and retention over time^11,27^. The development of more efficacious training schedules will support optimal delivery in terms of patient adherence and service cost and will be useful when combining cognitive training with interventional treatments such as brain stimulation or cognitive enhancing drugs. This may also guide the frequency of an increasing number of on-line cognitive training offerings.

The aim of this study was to investigate two training regimens, intensive and daily, in individuals with schizophrenia using implicit language learning. Implicit language learning has the advantage of being robustly impaired in schizophrenia^16^, and is based on statistical probabilities of coupling information extracted from the environment^33^. Similar training has been shown to be effective in neurological disorders including in language reacquisition in aphasia after stroke^34^; and has been advocated in the treatment of neurobiological disorders such as dyslexia^35^. We hypothesized that implicit learning rates will be significantly higher in the daily training group, because there is greater opportunity for consolidation^17,36^.

## Methods

### Participants

Analyses were based on two independent studies investigating the pro-cognitive effects of non-invasive brain stimulation and modafinil in individuals with schizophrenia. A total of 47 participants across both studies were selected; 24 individuals from the sham group in the brain stimulation study^17^, and 23 individuals from the placebo group in the modafinil study^16^.

The inclusion criteria for both studies were similar. All participants met the DSM-IV criteria for schizophrenia or schizoaffective disorder, were clinically stable and on stable doses of antipsychotics for at least 4 weeks prior to baseline visits. Antipsychotic medications were not changed for any participant. All participants scored on Wechsler Test of Adult Reading (WTAR) within the normal range. Furthermore, the modafinil study required a score of less than 4 on the following items of the Positive and Negative Syndrome Scale (PANSS): conceptual disorganization, hallucinatory behavior, unusual thought content, all items on the negative subscale.

Both studies excluded participants taking hypnotics, those meeting the DSM-IV criteria for alcohol or substance dependence within the past 6 months and participants with a history of neurological disorders or significant head injury that occurred with a loss of consciousness or required hospitalization. Additionally, the tDCS study excluded participants with a current or past history of skin disease while the modafinil study excluded participants taking compounds with known pharmacokinetic interactions with modafinil.

Following screening, socio-demographic data was collected from participants in both studies. All study participants provided informed written consent. Study #1 was approved by the Stanmore National Research Ethics Committee (REC number 11/LO/0248) and study #2 was approved by the West London Research Ethics Committee 2 (REC number 10/H0711/14). All study procedures were conducted in accordance with the Declaration of Helsinki.

### Cognitive training schedule

**Figure 1:**
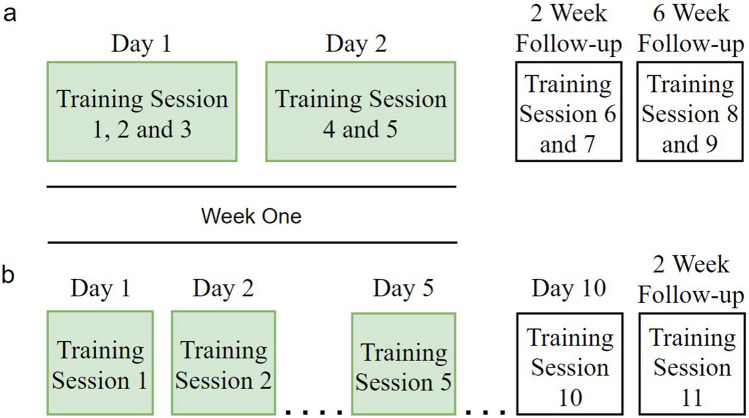
Cognitive Training Schedule for the daily group (**a**) and intensive group (**b**). Green background: cognitive training sessions included in the current analysis ce check ms and insert.

Study #1 investigated the effect of prefrontal transcranial Direct Current Stimulation (tDCS) along with cognitive training on learning in schizophrenia. Cognitive training sessions were conducted intensively with 45-min breaks between each session. The cognitive training sessions took place over 2 days, with three training session during day 1 and two the following day, separated by 45 min between-session breaks. During the 2 and 6 weeks follow-up visits two training sessions were administered^17^ (see Fig. 1a). 49 participants were recruited for study #1 and were randomly assigned into ‘active’ and ‘sham’ stimulation groups. Task data from the sham group were classified into the ‘intensive’ group.

Study #2 investigated the pro-cognitive effects of modafinil along with cognitive training in schizophrenia. The cognitive training session took place once a day, with ten sessions spread over consecutive weekdays (see Fig. 1b). A follow-up assessment was conducted 2 weeks after the end of the cognitive training.

48 participants were randomized to either ‘modafinil’ or ‘placebo’ groups. Participants from the placebo group were classified in to the ‘daily’ group. For the purpose of this study we compared learning rates between each session 1–5 from both studies.

### Cognitive training

Both studies assessed implicit learning using the same computerized language-learning task developed by Breitenstein and Knecht^37^. The task is based on the principle of associative learning, using a visual representation of a common object being paired with a novel pseudo-word (neologism). The task consisted of 50 black and white drawings presented along with 50 neologisms normalized in loudness and length (600 ms). Participants indicated if a picture-neologism pairing was “correct” (e.g., door and /bini/) or “incorrect” (e.g., door and /skog/), using the keyboard letters ‘K’ and ‘D’ respectively (Supplementary Fig. S1).

During each cognitive training session participants were presented with 400 pseudo-randomized picture-neologism pairings in two blocks of 200, separated by 90 s. The inter-stimuli interval was set at 1500 ms and the response time at 1000 ms. The inter-stimuli interval was set at 1500 ms and the response time at 1000 ms. Picture presentation commenced 200 ms after acoustic presentation of the neologism. The pace of the task was rapid by design to prevent participants consciously rehearsing stimuli. Each neologism and picture was repeated 4 times in each block. The randomization was such that the correct pairing of a given picture-neologism appeared twice in a block, and the same picture was paired incorrectly with two different neologisms. The incorrect pairing remained the same in the second block of the task but occurred only during one run of the cognitive training sessions. Therefore, the ratio of correct to incorrect pairing increased with each run; from 4:2 in session one to 8:2 in session two and then to 20:2 after five training sessions.

Participants were not given explicit instructions or made aware of the underlying objective of the learning task but were asked to respond if they thought that the neologism-picture pairings matched.

### Statistical analyses

Group differences in socio-demographic and clinical characteristics were assessed using t-tests for continuous and chi-squared tests for categorical variables.

Given the task design each response could be categorized in the following: (1) accurate indication that a picture and sound matched (true positive), (2) accurate indication that a picture and sound did not match (true negative), (3) false indication that a picture and sound matched (false positive), and (4) false indication that a picture and sound did not match (false negative).

Due to the task design (200 correct response and 200 incorrect responses in task run), if responses were made to all the stimuli, by chance participants would score 50% and any learning will increase this score. However, any missing responses add noise to this chance performance. Taking into consideration the fast pace of the task, and the nature of the clinical group, here a more sensitive outcome measure was sought. Adapted from machine learning, the Matthews correlation coefficient (MCC) provided a measure of true and false positives and negatives, making use of all the data and a better measure of learning in a dichotomous binary classification as defined within a four cell contingency table^38^. MCC summarizes fully all possible task responses. It takes a range from − 1 to + 1, where 0 indicates chance performance and values above are indicative of learning. In addition, its accuracy is independent of missing responses, yielding a balanced measure of learning and an easy comparison with respect to chance baseline performance^39^. The MCC was calculated for each training session based on the formula:

$$MCC = \frac{TP \times TN - FP \times FN}{{\sqrt {\left( {TP + FP} \right)\left( {TP + FN} \right)\left( {TN + FP} \right)\left( {TN + FN} \right)} }}$$

TP-true positive; TN-true negatives, FP-false positives, FN-false negatives.

Data analysis was conducted by specification of full maximum likelihood-random effect multilevel model (MLREM). We included the first five sessions from each study thus allowing for the assessment of an equal number of sessions completed within 1 week. The MLREM included the MCC outcome score from each training session 1 through 5 with fixed categorical effect of group (1-Daily, 0-Intensive) and time (1–5), and interaction of time (1–5) and group (1-Daily, 0-Intensive). We conducted an exploratory analysis of retention by comparing the follow-up score (2 weeks post-training) minus the final training session (session 5 for the intensive and session 10 for the daily group) using a two-sample t-test. Data analyses were conducted using STATA 15.1.

## Results

There were no significant differences in any of the baseline socio-demographic or clinical characteristic of the two groups (Table 1), except for antipsychotic medication dose.

**Table 1:** Clinical and socio-demographic information of participants.

	Intensive	Daily	t Value	*p* Value
Participants	n = 24	n = 23		
Age	38.7, SD 9.4	34.9, SD 9.8	1.37	0.18
Gender	4 females	7 females		0.90
Education	12.2, SD 2.4	12.4, SD 3.0	− 0.26	0.80
PANSS P	13.5, SD 4.4	12.6, SD 4.4	0.72	0.47
PANSS N	14.5, SD 5.4	15.3, SD 4.3	− 0.55	0.59
PANSS G	27.4, SD 7.6	27.3, SD 5.2	0.05	0.96
PANSS COMP	55.4, SD 14.3	55.2, SD 11.3	0.06	0.95
WTAR	97.0, SD 14.5	93.5, SD 18.7	0.62	0.54
Chlorpromazine Equivalent	520, SD 321	254.2, SD 195.3	3.48	0.001

*SD* standard deviation, education-in years, *PANSS* Positive and Negative Syndrome Scale (*P* positive, *N* negative, *G* general, *C* composite), *WTAR* Wechsler test of adult reading.

### Implicit learning

**Figure 2:**
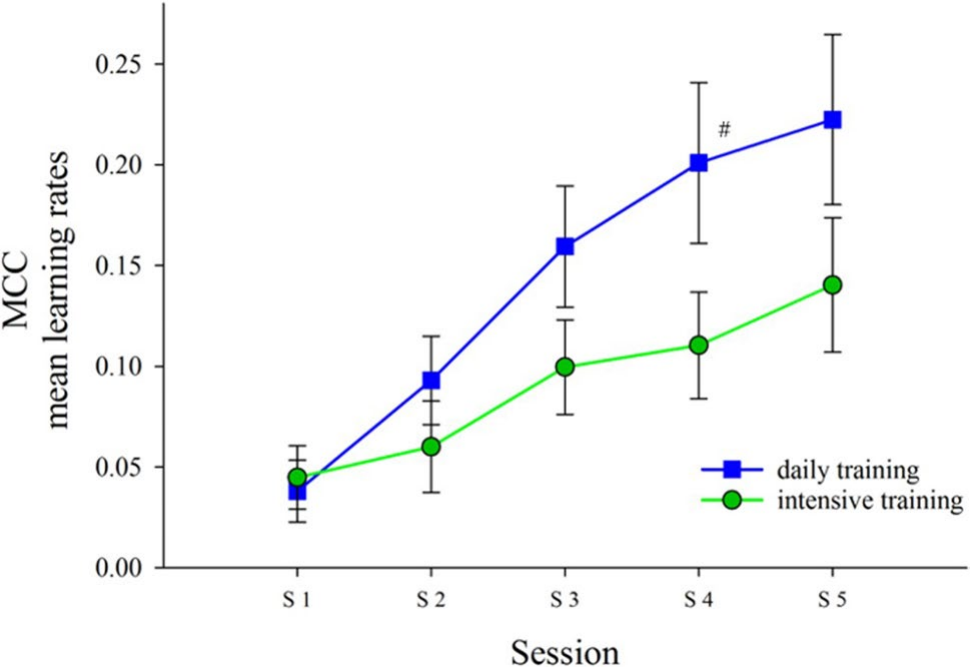
MCC mean learning rates over five sessions for both daily and intensive training regimens measured with MCC. *S* session, *MCC* Matthew’s correlation coefficient, higher values indicate better performance. # significant difference. Error bars represent standard errors.

There were no between group differences in MCC at session 1, (b = − 0.002, − 0.05 to 0.04, *p* = 0.93). The interaction of group and time was significant at session 4 (b = 0.08, 0.01–0.15, *p* = 0.03). The interaction of group and time was insignificant at session 2 (b = 0.02, − 0.02 to 0.07, *p* = 0.31), with a trend towards significance at session 3 (b = 0.08, − 0.01 to 0.11, *p* = 0.09) and 5 (b = 0.08, − 0.01 to 0.17, *p* = 0.08) (see (see Table 2 and Fig. 2).

**Table 2:** Behavioural performance in implicit learning as measured by MCC.

	b	95% CI	*p*
**Whole sample**			
Session 1	− 0.002	− 0.05 to 0.04	0.93
Session 2	0.02	− 0.02 to 0.07	0.31
Session 3	0.05	− 0.01 to 0.11	0.09
Session 4	0.08	0.01 to 0.15	0.03
Session 5	0.08	− 0.01 to 0.17	0.08

*CI* confidence interval, *b* beta coefficient of task performance during training sessions 1–5.

After controlling for age, IQ and dose of psychotic medication (chlorpromazine equivalents) there were no between group differences in MCC at session 1 (b = − 0.001, − 0.06 to 0.06, *p* = 0.97). The interaction of group and time was significant at session 4 (b = 0.08, 0.002–0.16, *p* = 0.04). The interaction of group and time was insignificant at session 2 (b = 0.03, − 0.02 to 0.09, *p* = 0.18); session 3 (b = 0.05, − 0.02 to 0.11, *p* = 0.15) ; and session 5 (b = 0.08, − 0.02 to 0.11, *p* = 0.11) ) (Table 3); neither IQ, age, nor chlorpromazine equivalents representing significant predictors: WTAR (b = 0.0005 − 0.001 to 0.002, *p* = 0.55); age (b = 0.0001, − 0.003 to 0.003, *p* = 0.94; chlorpromazine equivalent (b = 0.00002 − 0.00008 to 0.0001; *p* = 0.69). For model fit assessment see Supplementary Table T1.

**Table 3:** Behavioural performance in implicit learning as measured by MCC, controlled for age and IQ.

	b	95% CI	*p*
**Whole sample**			
Session 1	0.002	− 0.05 to 0.05	0.99
Session 2	0.02	− 0.02 to 0.07	0.31
Session 3	0.05	− 0.01 to 0.11	0.10
Session 4	0.08	0.01 to 0.15	0.02
Session 5	0.09	− 0.004 to 0.17	0.06

*CI* confidence interval, *b* beta coefficient of task performance during training session 1–5.

### Exploratory analysis

Three participants’ data were missing at the follow up, or last session. There were no significant between group differences in retention measured with MCC (t_(1,42)_ = 0.29, − 0.1 to 0.08, *p* = 0.77).

## Discussion

This is the first study to investigate the effects of training intensity on performance and retention of implicit language learning in individuals with schizophrenia. We utilized a task developed by Breitenstein and Knecht (2002), which allows for the acquisition/retrieval of knowledge without explicit instructions or feedback^40^, and correlates with performance in a variety of cognitive domains, including verbal memory^37^.

In keeping with prior research our study found that individuals with schizophrenia are able to learn using implicit processes, as participants’ performance improved across all the five training sessions. Confirming our hypothesis, the daily group demonstrated increased performance compared to the more intensive group, but only at session 4. This indicates that daily training is advantageous in comparison to the more intensive training, over a short period.

The main difference between the two regimen is the opportunity for more regular consolidation in the daily training condition. Learning and memory have been linked to consolidation processes, specifically it has been demonstrated that perceptual learning of synthetic speech is enhanced by sleep; facilitating the recovery and retention of material learnt opportunistically at any time of the day^41^. In the intensive group, the first three training sessions took place on the same day, thus this group had increased training time. Interestingly, it seems that the consolidation period had a somewhat differential effect on learning; whereby in the daily group we observed a steady increase in learning rates, the consolidation resulted in significantly worse performance in the intensive group. However, while sleep is the most prominent factor in consolidation between these different training schedules; we did not explicitly control for variation in sleep over the training period and it is possible that other variables may also have been involved in improving consolidation in the daily session group. These may have included increased fatigue or decreased motivation accompanying increased frequency of training^42^. Differential effects of training intensity on learning have been observed in another study in healthy subjects, Feld et al.^43^ demonstrated that sleep supported memory consolidation depends on the number of items to be learned. They found that sleep-related consolidation improved performance when learning 160 word-pairs, however had no effect on learning when 320 word-pairs were presented, suggesting that with increasing load of information sleep does not favor consolidation processes, rather it may favor forgetting processes.

Whereas due to methodological differences our study cannot directly answer the question if consolidation is affected by the amount of information processed, it is nonetheless encouraging that individuals with schizophrenia display a similar pattern of learning processes as healthy control, albeit at a slower rate, as demonstrated in the modafinil study^16^. This lower learning might potentially be a consequence of reduced sleep spindle activity associated with synaptic plasticity observed in schizophrenia^44^. Certainly, in our previous work to improve cognition^17^, there was an important role posited for consolidation effects using tDCS. Interestingly, efforts to enhance sleep-dependent consolidation by using tDCS during sleep resulted in a significant increase in retention of verbal material as well as improved mood in individuals with schizophrenia^45^. Taken together these results suggest that maximizing the effectiveness of a cognitive intervention for schizophrenia should involve consideration of sleep effects on learning alongside an optimized training regimen.

Cognitive training, particularly on-line, is a burgeoning area of research in schizophrenia, with much of interest in the benefits of regular training and impact on retention of performance following training. Impaired retention is schizophrenia is a known issue, and increasing performance in a lasting manner is the main goal of training^46^. Unfortunately, we were not able to examine that in this study directly, because of the differing numbers of training sessions between the two regimens. An exploratory examination of 2-weeks retention did not differ between the regimens, although the daily training had completed ten rather than the five sessions in the intensive regimen.

While a daily training regimen appears to be somewhat more beneficial when investigating cognitive enhancers, attrition rates in cognitive rehabilitation programs for individuals with schizophrenia and schizoaffective disorder reach as high as 37%^47^ making such routines more difficult to implement in clinical practice. Thus, when motivation to attend training is low or when service provision supports more intensive training, an intensive training regimen is viable if possibly less effective.

### Limitations and future research

This study is limited by the differing objectives of the studies from which the data was used as it limits the points of comparison^11^. This is compensated for in part by the sample size. The absence of functional measures at follow-up limits assessment of functional changes. However, as we were primarily interested in understanding the effects of training intensity on rate of learning, our study design was centered around available data for limited comparable sessions over a shorter period. The differences in the cognitive training regimen may have affected measured outcomes but this limitation is somewhat mitigated by the fact that training sessions in both studies lasted for ~ 60 min and aimed to improve similar cognitive processes. We also observed a significant between group difference in chlorpromazine equivalents. However, medication dosage was not a significant outcome predictor, as demonstrated in our analysis. Additionally, the task chosen for this study did not rely on explicit cognitive strategies and effortful learning.

Furthermore, tailoring the difficulty of the cognitive training to the participant would enable them to get the most out of the training and set a higher benchmark in cognitive remediation. Use of alternative implicit learning tasks may have led to more desirable performance increases but potentially risked more elements of conscious processing taking place. Serial reaction time tasks, dynamic system control tasks and artificial grammar learning tasks are examples of commonly used implicit learning trials, however, these tasks may not be limited to the use of purely implicit domains. Some participants can become aware of the learning aims and therefore be employing explicit cognitive processes during the task^48,49^. Of note is that the actual session 3 and 5 between group learning data are not significant—this could be due to a lack of power or due to the session 4 data being an incidental outlier—we suggest that the plot in Fig. 2 is strongly supportive of the former conclusion.

We suggest that future studies include other implicit and explicit learning training tasks, which may improve performance gain and generalizability effects.

### Conclusion

We have demonstrated that there is a significant performance gain in an implicit learning task when it is performed daily rather than more intensively. Our results suggest that intensive training may provide a viable but less effective alternative in clinical practice, when daily attendance is impractical or unattainable.

## Supplementary Information

Supplementary Information.
