# Author Correction: The effect of training intensity on implicit learning rates in schizophrenia

**DOI:** 10.1038/s41598-021-92493-5

**Published:** 2021-06-15

**Authors:** Natasza D. Orlov, Jessica Sanderson, Syed Ali Muqtadir, Anastasia K. Kalpakidou, Panayiota G. Michalopoulou, Jie Lu, Sukhi S. Shergill

**Affiliations:** 1 Cognition Imaging Schizophrenia Lab, Department of Psychosis Studies, Institute of Psychiatry Psychology and Neuroscience, King’s College London, London, UK; 2 Harvard Medical School, Athinoula Martinos Center for Biomedical Imaging, Massachusetts General Hospital, Boston, USA; 3 Department of Radiology, Xuanwu Hospital, Capital Medical University, Beijing, China; 4 Precision Brain Imaging Lab, Department of Neuroscience, Medical University of South Carolina, Charleston, USA; 5 Lahore University of Management Sciences, Lahore, Pakistan; 6 Marie Curie Palliative Care Research Department, University College London, London, UK

Correction to: *Scientific Reports* 10.1038/s41598-021-85686-5, published online 22 March 2021

The Funding section in the original version of this Article was incomplete.

“The authors of this study report no conflicts of interest, financial or otherwise. No conflicts of interest were reported in study #1. Study #2 reported conflicts of interests for three authors which are detailed in that publication.”

now reads:

“The authors of this study report no conflicts of interest, financial or otherwise. No conflicts of interest were reported in study #1. Study #2 reported conflicts of interests for three authors which are detailed in that publication. This study represents independent research part funded by the National Institute for Health Research (NIHR) Maudsley Biomedical Research Centre at South London and Maudsley NHS Foundation Trust and King’s College London. The views expressed are those of the authors and not necessarily those of the NHS, the NIHR or the Department of Health and Social Care.”

The original Article has been corrected.

