# Supplementary material for: The effect of training intensity on implicit learning rates in schizophrenia
Source: Sci Rep. 2021 Mar 22;11:6511. doi: 10.1038/s41598-021-85686-5 (PMC7985318; doi:10.1038/s41598-021-85686-5)
Supplement: Supplementary file 1 — Supplementary Information. [file 41598_2021_85686_MOESM1_ESM.docx]

# The Effect of Training Intensity on Implicit Learning Rates in Schizophrenia

# Natasza D. Orlov^1,2,3,4, +,^ *(natasza.orlov@kcl.ac.uk), Jessica Sanderson^1,+^ (jessica-rose@live.com), Syed Ali Muqtadir^1,5,+^ (samuqtadir@protonmail.com), Anastasia K. Kalpakidou^1,6^ (a.kalpakidou@ucl.ac.uk), Panayiota G. Michalopoulou^1^ (panayiota.michalopoulou@kcl.ac.uk), Jie Lu^3,^* (imaginglu@hotmail.com), , Sukhi S. Shergill^1^ (sukhi.shergill@kcl.ac.uk)

# ^1^ Department of Psychosis Studies, Cognition Imaging Schizophrenia Lab, Institute of Psychiatry Psychology and Neuroscience King’s College London, UK

# ^2^ Harvard Medical School, Athinoula Martinos Center for Biomedical Imaging, Massachusetts General Hospital, Boston, USA

# ^3^ Department of Radiology, Xuanwu Hospital, Capital Medical University, Beijing, China

# ^4^ Department of Neuroscience, Precision Brain Imaging Lab, Medical University of South Carolina, Charleston, USA

# ^5^ Lahore University of Management Sciences, Lahore, Pakistan

# ^6^ Marie Curie Palliative Care Research Department, University College London, UK

# * Corresponding author(s)

# + Authors contributed equally

**Corresponding author(s) contact details:**

Dr. Natasza D. Orlov

Lab of Precision Neuroimaging, Department of Neuroscience, Medical University of South Carolina, 135 Canon Street, Charleston, 29 425, USA; Tel number: +1 781 957 8145

Dr. Jie Lu

Department of Radiology, Xuanwu Hospital, Capital Medical University, Beijing, China; Tel number: +86-010-83911305

**Supplementary Figure 1.** Task design.

**
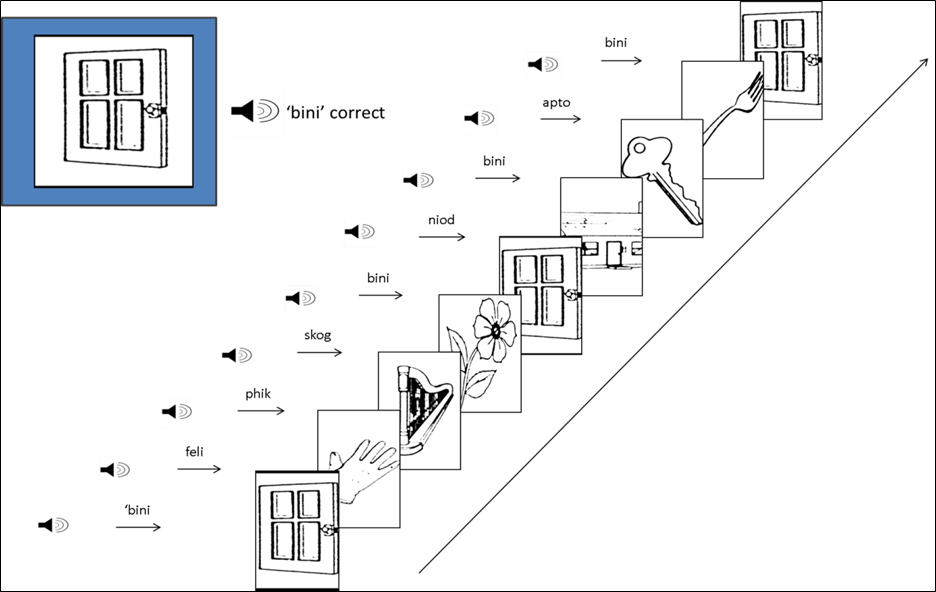
**

Comparison between the original score and MCC below a study participant’s performance during the first task run:

A participant responding ‘not correct pairing’ to stimuli with an overall 64 missing would get a 43% score based on the correct response only, whereas the MCC puts this score at 0-still indicating chance performance.

**Supplementary Table T1.** Model fit assessment

| Model | AIC | BIC |
| --- | --- | --- |
| Basic | -510 | -465 |
| Age | -508 | -460 |
| WTAR | -471 | -423 |
| Chlorpromazine equivalent | -469 | -417 |
| Age + WTAR | -469 | -418 |
| Age + WTAR+ chlorpromazine equivalent | -427 | -370 |

AIC: Akaike Information Criterion, BIC: Bayesian Information Criterion

**Additional Analysis**

The regression analysis on combined sessions 3, 4 and 5 yielded the following results: t(1,45) = 1.73, p = 0.09. The regression analysis of combined session 4 and 5 yielded the following: t(1,46) = 1.63, p = 0.11.

We ran a full maximum likelihood-random effect multilevel model (MLREM) on the average of incorrect and correct responses after an incorrect response and found no significant between group differences.

There were no between group differences in incorrect response after correct associations at any time point: session 1, (b=0.87, -9.2-10.9, p=0.87); the interaction of group and time was insignificant at session 2 (b=3.21, -7.4-13.8, p=0.55), at session 3 (b=10.2, -2-16.1, p=0.69), at session 3 (b=-10.8, -27.4-5.8, p=0.20), as well as session 5 (b=-3.37, -23.5-16.9, p=0.75).

There were no between group differences in incorrect response after incorrect associations at any time point: session 1, (b=0.87, -10.6-8.8, p=0.86); the interaction of group and time was insignificant at session 2 (b=-3.09, -14.2-8.1, p=0.59), at session 3 (b=10.3, -2.7 - 23.2, p=0.12), at session 4 (b=-10.8, 5.4 - 26.9, p=0.19), as well as session 5 (b=1.3, -18.4 – 21.1, p=0.89).

The mean % of correct and incorrect associations for each group and session (Supplementary Table T2 and T3) should now serve to facilitate comparison with previous reports (1-2), including two papers from our groups (3-4).

**Supplementary Table T2.** Percentage of correct responses over the course of five training sessions

| **Session** | **Intensive** | **Daily group** |
| --- | --- | --- |
| **1** | 48.3 | 47.6 |
| **2** | 48.4 | 47.5 |
| **3** | 51.6 | 49.8 |
| **4** | 51.5 | 52.4 |
| **5** | 53 | 56.5 |

**Supplementary Table T3.** Percentage of incorrect responses over the course of five training sessions

| **Session** | **Intensive** | **Daily group** |
| --- | --- | --- |
| **1** | 51.7 | 52.4 |
| **2** | 51.6 | 52.5 |
| **3** | 48.4 | 50.2 |
| **4** | 48.5 | 47.6 |
| **5** | 47 | 43.5 |

**References**

1. Breitenstein, C., & Knecht, S. (2002). Development and validation of a language learning model for behavioral and functional-imaging studies. *Journal of neuroscience methods*, *114*(2), 173-179.
2. Flöel, Agnes, et al. "Noninvasive brain stimulation improves language learning." *Journal of cognitive neuroscience* 20.8 (2008): 1415-1422.
3. Gilleen, J., Michalopoulou, P. G., Reichenberg, A., Drake, R., Wykes, T., Lewis, S. W., & Kapur, S. (2014). Modafinil combined with cognitive training is associated with improved learning in healthy volunteers–a randomised controlled trial. *European Neuropsychopharmacology*, *24*(4), 529-539.
4. Michalopoulou, P. G., Lewis, S. W., Drake, R. J., Reichenberg, A., Emsley, R., Kalpakidou, A. K., ... & Applegate, E. (2015). Modafinil combined with cognitive training: pharmacological augmentation of cognitive training in schizophrenia. *European Neuropsychopharmacology*, *25*(8), 1178-1189.
